# QR code technology in individual identification training provides an introduction in hands-on forensic DNA genotyping to medical students

**DOI:** 10.1186/s12909-023-04284-3

**Published:** 2023-05-05

**Authors:** Li Lai, Li Liu, Yaochen Wang, Shanlong Liu, Jiancheng Zhang, Xiaochun Zheng

**Affiliations:** 1 Provincial Clinical College of Fujian Medical University, No.134 East Road, Fujian, Fuzhou, 350001 China; 2 Central Laboratory of Fujian Provincial Hospital, No.134 East Road, Fujian, Fuzhou, 350001 China

**Keywords:** QR code, Hands-on, Individual identification, Education, Training

## Abstract

**Background:**

Forensic biology is a subject in the field of forensic science that stresses practical teaching and training in laboratory skills. Visualization of deoxyribonucleic acid (DNA) profiles is important in individual identification and is easily performed by well-trained examiners. Therefore, developing a novel training project for obtaining individual DNA profiles can improve the quality of teaching for medical students or trainees. DNA profiles based on quick response (QR) codes can also be applied to practical teaching and operation training for individual identification.

**Methods:**

A novel training project was developed through an experimental course in forensic biology. Blood samples and buccal swabs with oral epithelial cells, as used in the forensic DNA laboratory, were obtained from medical students at Fujian Medical University. DNA was isolated, and a number of short tandem repeat (STR) loci were used as genetic markers to generate DNA profiles. The students converted DNA profiles and individual information into a QR code. The QR code could then be scanned by a mobile phone for consulting and retrieval. Gene identity cards with QR codes were produced and provided to every student. The participation rate and passing rate of students who participated in the novel training project were calculated and compared with those of students in the traditional experimental course, and a chi-square test was carried out by SPSS 23.0 software to evaluate the teaching effectiveness. *p* < 0.05 indicated significant differences. In addition, a survey was conducted to investigate the likelihood of using of gene identity cards with QR codes in the future.

**Results:**

A total of 54 of 91 medical students who studied forensic biology participated in the novel training project in 2021. Only 31 of 78 students who studied forensic biology participated in the traditional experimental course in 2020. The participation rate in the novel training project was 24% higher than that of the traditional experimental course. The participants in the novel training project showed better performance in forensic biological handling techniques. The passing rate of the students in the forensic biology course with the novel training project was approximately 17% higher than that of the students in the former course. The participation rates and passing rates of the two groups were significantly different (*χ* = 6.452, *p* = 0.008 and *χ* = 11.043, *p* = 0.001). In the novel training project, all participants made 54 gene identity cards with QR codes. Furthermore, in the DNA profiles of four African students who participated, we found two rare alleles that were not discovered in Asians. The survey showed that the use of gene identity cards with QR codes was accepted by most participants, and the likelihood of future utilization was 78%.

**Conclusion:**

We established a novel training project to promote the learning activities of medical students in experimental forensic biology courses. The participants showed great interest in using gene identity cards with QR codes to store general individual identity information and DNA profiles. They also examined the genetic population differences between different races based on DNA profiles. Hence, the novel training project could be useful for training workshops, forensic experimental courses, and medical big data research.

## Background

Forensic biology is the study of biological materials related to the human body in medico-legal cases for the purpose of providing scientific evidence. It involves multiple disciplines, such as medicine, laboratory work, genetics, chemistry and biochemistry [1]. At present, individual identification using DNA profiles is the main task of forensic biology with respect to human identification.

Only a few medical universities offer forensic biology courses to medical students in China. Furthermore, fewer experimental courses on forensic biological techniques can be carried out because of the limitations of laboratory space and instruments. The traditional teaching mode of forensic biology is mainly lectured by teachers; hence, medical students lack interest and motivation in learning [2]. Because the mastery of forensic biological techniques must be based on a large amount of practical training, it is important to carry out experimental courses on forensic biological techniques in medical universities.

An early interview survey of medical students at Fujian Medical University showed that these students had a strong willingness to gain hands-on experience with forensic biological techniques. To cultivate more well-trained students, we created a novel training project for the visualization of DNA profiles and bioinformation conversion by QR codes. The novel training project was designed to be an upgraded version of the traditional experimental course.

As information carriers, QR codes are currently widely used, and they can be easily scanned by a mobile terminal. They have the advantages of a high recognition rate, a large amount of stored information, low cost and simple operation [3]. Because of these advantages, QR codes have also been considered a solution for COVID-19 information overload during the epidemic [4].

Our aim was to create a novel training project that will help medical students master the theoretical knowledge of forensic biology and perform the common technique of DNA profiling. We also expect that this will increase medical students’ learning interest and inspire them to fully participate in hands-on experimental courses, eventually helping them to apply their knowledge.

## Methods

### Participants

At Fujian Medical University, a forensic biology course was offered as an elective undergraduate course to both native students and international students. Overall, 78 medical students from the classes of 2018 and 2019 chose to take the course in 2020, and 31 of them were willing to participate in the traditional experimental course; these students were used as the control group.

A novel training project was offered in 2021 as an upgrade to the experimental course. Ninety-one medical students from the classes of 2020 and 2021 chose to take the course, and 54 of them were willing to participate in the novel training project. The two groups of students received the same theoretical teaching content, and the teachers used the same teaching skills to complete the theoretical teaching task. Of note, four international students from Africa enrolled in the course.

### Study steps

**Fig. 1:**
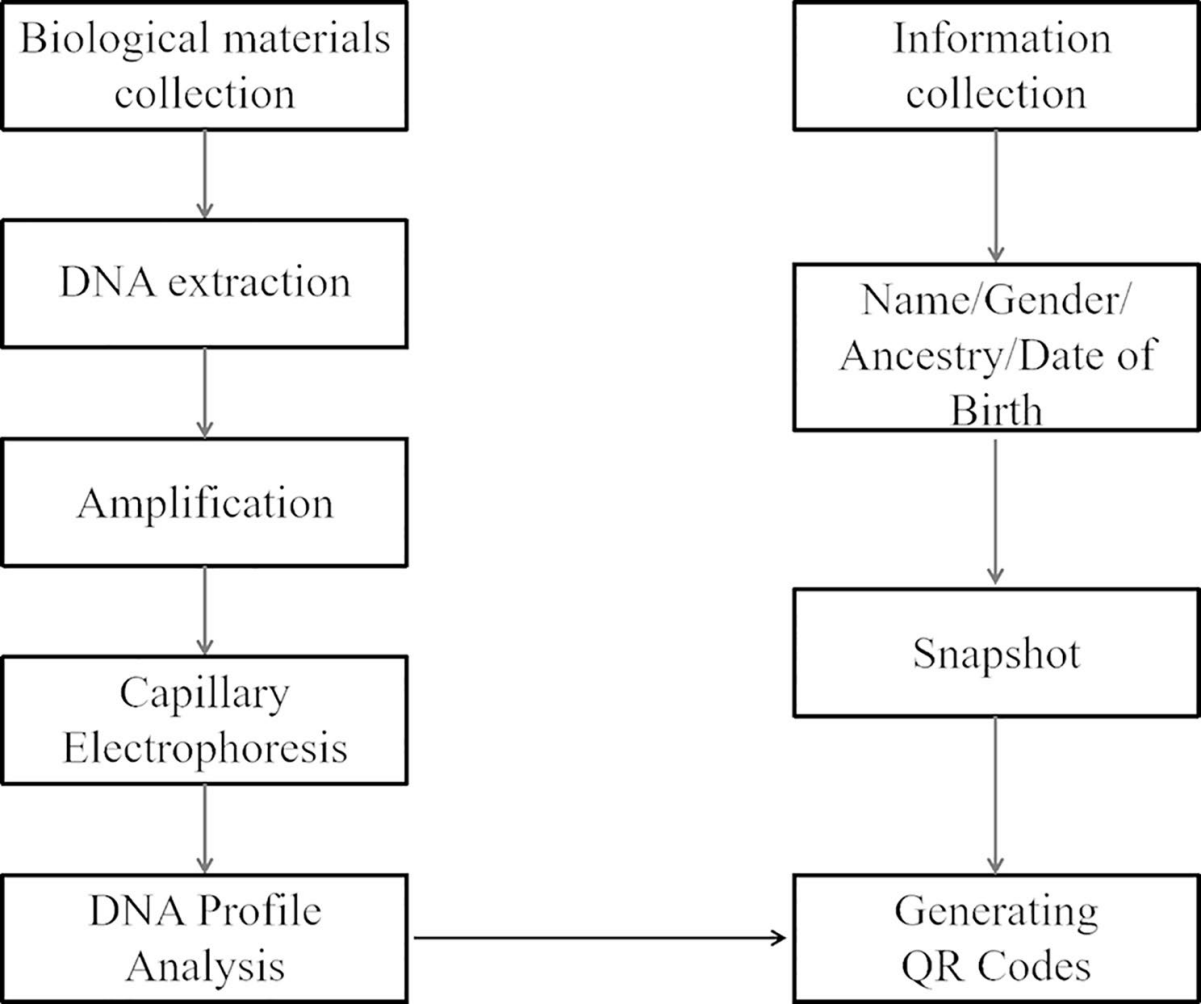
Study procedures of the novel training project

The project was composed of two phases of practical application. Phase A concerned the development of DNA profiles, and Phase B involved the creation of QR codes. Making DNA profiles included five steps (i.e., biological material collection, DNA extraction, amplification, capillary electrophoresis and DNA profile analysis). The QR codes contained not only the common individual information (e.g., name, gender, ancestry, and identity number) but also DNA profiles. The entire procedure was conducted by the medical students themselves with instruction by monitors. Figure 1 illustrates the overall study procedures of the novel training project.

The details of generating DNA profiles in a forensic DNA laboratory along with the details of creating QR codes are described below. After the project was completed, the participation rate for the novel training project was calculated and compared with that of the traditional experimental course. The passing rate of the students in the forensic biology course with the novel training project was also compared with that of the students in the traditional course.

### Sample collection

In the project, we allowed the participants to collect two types of biological samples at their discretion. Before collecting biological samples, 75% alcohol was sprayed on the collection site for disinfection, and the specimens were subjected to ultraviolet radiation for 30 min.

First, the participants completed the individual information registration forms, including their names, genders, nationalities and signatures. The left thumbprints and right thumbprints of participants were also recorded as individual-specific markers.

Bodily fluid (blood) and buccal (cheek) samples were collected with sterile cotton swabs by participants as experimental materials for the training experiment. A finger prick was used to collect the blood sample, and a sterile swab was used to collect the buccal cells sample.

The procedure for a buccal swab was relatively simple. A person removed the swab from a sterile package and rubbed the cotton tip against the inside of another person’s cheek. The swab was then placed in a sterile container and sent to a laboratory for examination.

The biological samples were transferred to an FTA® Micro Card, labelled with the subject’s name, initials of the person collecting the sample, and date of collection, for storage purposes. The FTA® Micro Card was allowed to dry completely (30–60 min) at room temperature.

### DNA extraction

For the DNA extraction process, both the blood and the buccal sample were transferred to an Eppendorf (EP) tube, and sample preprocessing was performed using TE (Tris-EDTA) solution. Each sample was centrifuged at the maximum speed for 2 min at room temperature. Next, the supernatant of the lytic product was discarded, and 300 µl of 5% Chelex-100 solution was added to the sediment. The mixture was incubated at 56 °C for 30 min and held at 100 °C for 8 min to release double-stranded DNA.

### DNA amplification and detection

In the novel training project, we used a commercial STR kit to obtain individual DNA profiles, which included 20 STR loci and amelogenin. DNA amplification was carried out in a 25-ml reaction volume with a PowerPlex 21 kit (Promega, Madison, WI, USA). The amplification reactions generally contained 0.5-1 ng DNA in 25 ml and used 28–30 cycles as described in the PowerPlex 21 kit technical manual.

Individual DNA profiles were generated by using a PowerPlex 21 kit and performed using the recommended procedures. DNA fragments in the amplification products were separated and detected on a 3130 Genetic Analyzer per the manufacturer’s instructions and analysed using GeneMapper ID v3.2 software.

### Creating and scanning QR codes

According to the hands-on design of the experimental course, we allowed the participants to record individual information (e.g., name, gender and nationality) by themselves. We also instructed them to convert the individual information and DNA profiles into a QR code by using an online QR code generator [5] at the end of the hands-on experimental course. The QR codes could be easily scanned by a mobile terminal, and the converted information could be directly obtained.

### Efficacy evaluation

In this study, we focused on whether the novel training project was better than the traditional experimental course. The participation rate and passing rate of the medical students in the two groups were calculated, and the chi-square test was carried out by SPSS 23.0 software to evaluate the differences in teaching effectiveness. Categorical data were analysed with the chi-square test. Differences were considered statistically significant if *p* < 0.05.

The same assessment for evaluating the passing rates was used in both groups. The monitors used the same theory teaching content and gave the same examination paper to the medical students in both groups. A survey was conducted among the students who participated in the novel training project to investigate the likelihood of utilization of gene identity cards with QR codes.

Due to the participation of students from different ethnic groups, we expected to discover some rare alleles or unique genotypes in the DNA profiles that could reveal genetic polymorphisms or genetic diversity to the participants.

### Hazards and safety precautions

Any hazards and safety precautions were covered by the safety regulations of Fujian Provincial Hospital and Fujian Medical University. Material Safety Data Sheets (MSDSs) were available from the chemical suppliers for chemical safety and exposure information. The proper laboratory safety guidelines for the forensic DNA laboratory at Fujian Provincial Hospital reduced or eliminated any safety issues involved in handling chemicals or glass slides, operating laboratory equipment and disposing of waste.

## Results

The numbers and participation rates of the two groups of medical students are shown in Table 1. There was a significant difference in the participation rates of the two groups (χ = 6.452 and *P* = 0.008).

**Table 1:** Differences in the participation of the two groups of medical students

	Number of students		*P* value
	Traditional course Novel training project		
Participated	31	54	* χ* = 6.452 *P* = 0.008
Did not participate	47	37	
Participation rate	35%(31/78)	59%(54/91)	

The participants in the novel training project showed better performance of forensic biological techniques. The teaching effectiveness was evaluated by analysing the passing rates of the two groups. Based on the scores of the final examination in Forensic Biology, the students in the forensic biology course with the novel training project had a 94.5% passing rate. In contrast, the students in the control group in the traditional experimental course had a 76.9% passing rate. The numbers and passing rates of the two groups of medical students are shown in Table 2. There was a significant difference in the passing rates of the two groups (χ = 11.043 and *P* = 0.001).

**Table 2:** Differences in teaching effectiveness of the two groups of medical students

	Number of students		*P* value
	Traditional course Novel training project		
Failed	18	5	*χ* = 11.043 *P* = 0.001
Passed	60	86	
Passing rate	76.9% (60/78)	94.5% (86/91)	

**Fig. 2:**
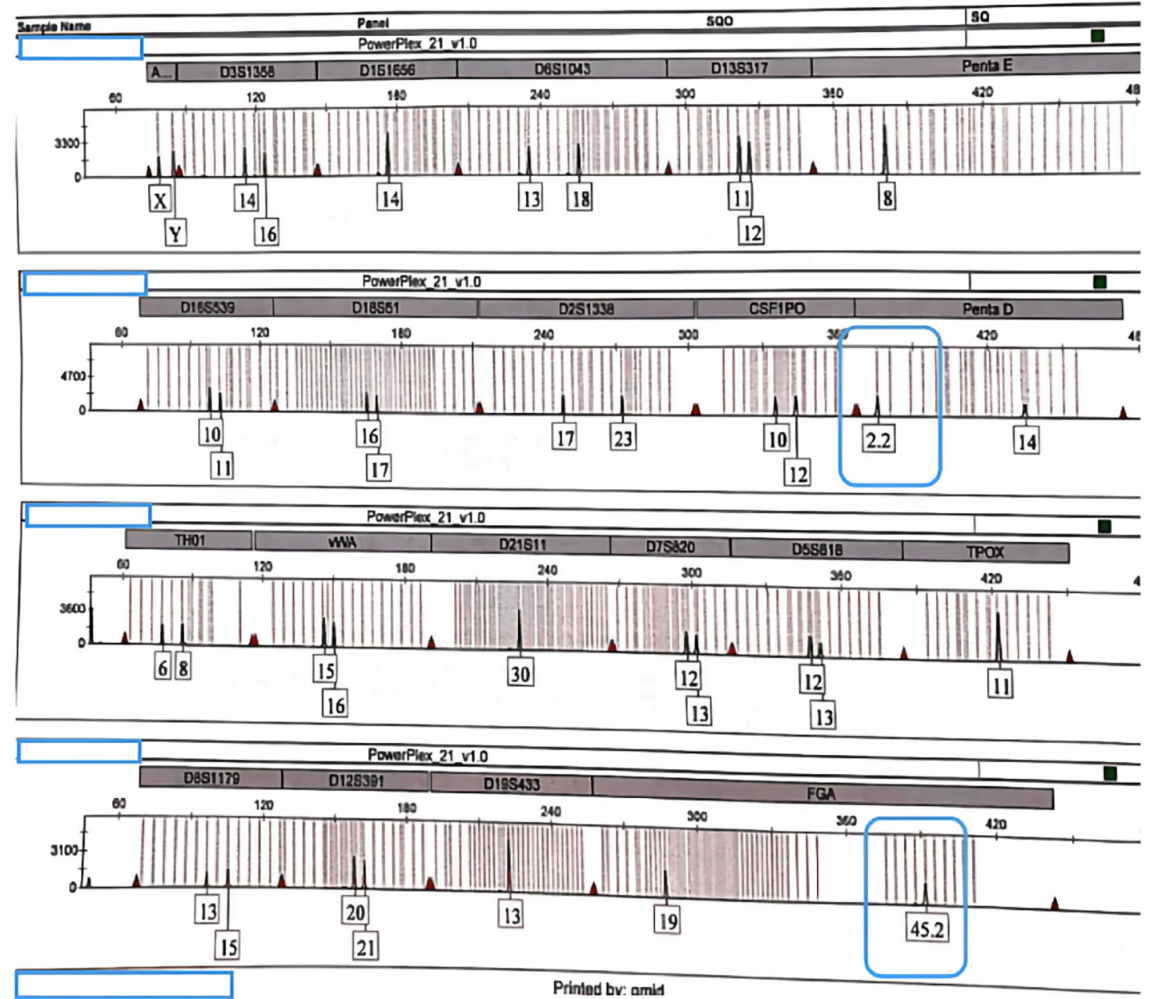
A Nigerian student’s DNA profile (red blocks indicate rare alleles)

In the novel training project, the participants created 54 gene identity cards with QR codes. Furthermore, an overseas student from Nigeria found two rare alleles in his DNA profile that had not been previously discovered in Asians. Figure 2 shows the DNA profile of the Nigerian student. Two rare alleles, allele 2.2 in Penta D and allele 45.2 in FGA, were discovered in the DNA profile.

**Fig. 3:**
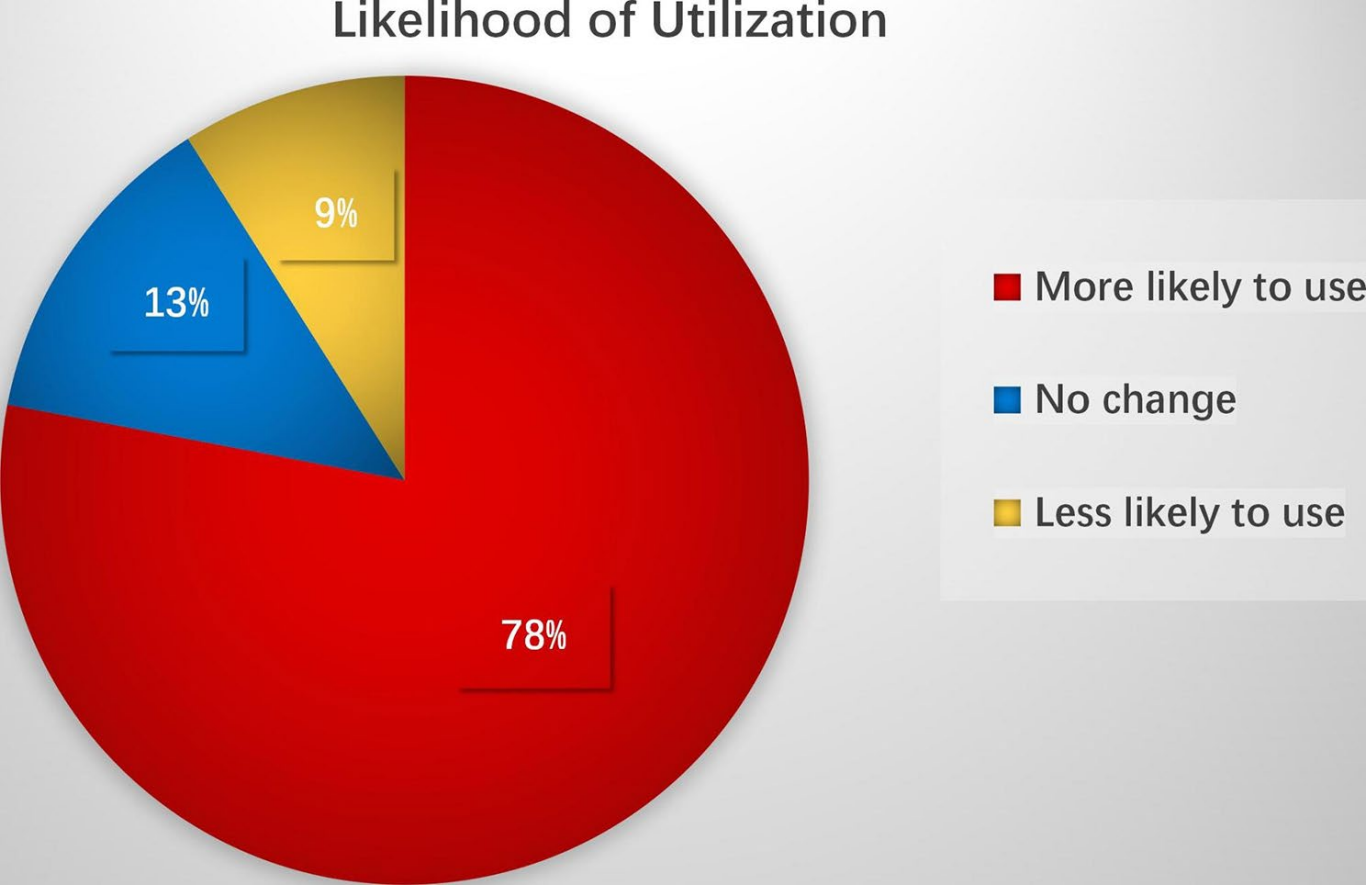
Survey results in percentages

After the novel training project was completed, a survey was administered to evaluate the likelihood of utilization of the gene identity card with QR codes. The survey was distributed to 54 participants, including 32 men and 22 women. The results showed that the gene identity cards with QR codes were accepted by most participants, and the expected likelihood of utilization was up to 78%. Forty-two participants showed a willingness to use gene identity cards with QR codes, but 7 participants thought that there was no change in the experiment course regardless of the information representation model. The other 5 participants expressed their concern about human rights and privacy policies. Figure 3 shows the likelihood of utilization of gene identity cards with QR codes reported by the participants.

As an experimental achievement, the gene identity cards with QR codes were produced with card printers. On the front side of the card, a snapshot of the participant and the QR code were shown clearly. Other individual information (e.g., name, sex, date of birth, nationality and identity number) was also present on the gene identity cards.

## Discussion

Currently, few universities in China offer an overall forensic medicine course, especially forensic biology, an important branch of forensic medicine that depends on advanced instruments and the knowledge of molecular genetics.

At Fujian Medical University, we offer a forensic biology course consisting of theoretical courses and a hands-on experimental course. The theoretical courses were so uninteresting that the students found it difficult to master the essence of the courses. Furthermore, the students had little opportunity for hands-on forensic practice, and because of the inactive teaching model, the students lost their enthusiasm.

Hence, we aimed to apply a more positive approach to enhance the students’ interest.

To achieve this, we created a novel training project that was an upgrade to the hands-on experimental course. In the project, in accordance with current technology, we integrated the visualization of DNA profiles and data conversion.

We found that the participation rate in the novel training project was 24% higher than that in the traditional course. There were significant differences in the participation rates of the two groups (*P* < 0.05).

It seemed that more students had interest in the novel training project and had strong intentions to participate.

Because the students in both groups were given the same examination paper, we can compare the learning effects of the two groups. The passing rate of the students in forensic biology course with the novel training project was 17% higher than that of the students in former course. There were significant differences in the passing rates of the two groups (*P* < 0.05). It seemed that the teaching effectiveness was improved after the novel training project was completed.

The survey about the utilization of QR codes indicated that most students appreciated the novel approach of individual identification and the integration of biotechnology and digital information technology. The novel approach seemed to have great application values in the field of individual identification.

With the promotion of teaching models, many education researchers have applied several advanced techniques to their teaching practices to enhance the effectiveness of teaching skills [6–9]. Multidisciplinary integration in education makes theoretical teaching easier to understand and makes practical teaching more vivid.

In this study, we present a novel training project combining the generation of DNA profiles and QR codes for the improvement of teaching models. QR codes are carriers of digital information and are currently widely used. QR codes can be easily created and scanned by a mobile terminal. They have the advantages of a high recognition rate, a large amount of stored information, low cost and simple operation. In the field of education, some educators have applied QR codes to improve the teaching effectiveness of various disciplines or have regarded QR codes as an important teaching tool. Most educators believe that QR codes are useful for exciting and engaging the students, especially in subject learning [3, 10–15].

According to the results of DNA profiles obtained in the novel training project, we discovered two rare alleles (e.g., allele 2.2 in Penta D and allele 45.2 in FGA) in the DNA profile of a Nigerian student. These rare alleles have not been reported in relevant literature about Chinese human gene resources.

After searching the NIST STRBase database [16], we found that the frequency of allele 2.2 in Penta D was 0.0430 in a total of 1036 Americans, including 0.1140 in 342 Afro-Americans, 0.0042 in 361 Caucasians, 0.0170 in 236 Hispanic or Latino Americans and 0 in 97 Asian-Americans [17]. The frequency of allele 45.2 in FGA was not supplied in NIST STRBase. A thorough survey revealed that the long (> 33) FGA alleles appear exclusively in populations of sub-Saharan Africans, Caribbean individuals of African descent, non-African Arabs and individuals of non-African Arab descent [18].

These findings suggest the existence of racial differences at the molecular level and helped the students more easily comprehend these differences. We are pleased to have discovered these rare alleles so that the students will show more interest in the course and in trying to discover the genetic differences among themselves.

As human biological materials and personal private information were used in the novel training project, we emphasized the protection of personal information. To convert a model for visualization of a DNA profile to a non-visual model, we chose the QR code as a digital-data storage medium that could be used to avoid data theft. With regard to this privacy issue that concerned some students, we upgraded security by establishing a database and web server used to store the individual information. The students could register a username and set up a password for the database so that they could scan QR codes to obtain a pathway to access the database, and they could also set up authorisation to browse their own individual information on the web server. We applied for a patent based on the security upgrade, and the patent was granted in 2021 (Grant Number CN213910234U) by the China National Intellectual Property Administration.

## Study limitation(s)

This study has some limitations. First, the assignment of medical students was not entirely random, because only the students who were willing to participate were included.

The varied demographic characteristics of the medical students could factor into the perception of QR code technology and its usage. Certain demographic factors could affect the perception, such as gender. The male students showed more willingness to use QR codes than the female students. The medical students who were familiar with basic biochemistry lab procedures and had hands-on experience could also affect the perception of DNA profiles.

Second, generating DNA profiles is time-consuming, and the major precision instruments involved require authorization before use. The procedures of PCR amplification and capillary electrophoresis were performed by the instructors. This decision was made because the operations would take longer if they were conducted by students themselves, and unexpected reagent consumption would increase.

## Conclusion

Multidisciplinary integration has been involved in an increasing number of experimental courses and practical training projects. Students will benefit from multidisciplinary integration to master various skills in the learning stage.

With the creation of the novel training project, the multidisciplinary integration teaching model allowed the medical students to (1) show their interest in participating in the novel training project in forensic biology, (2) gain a deep understanding of the differences in human DNA phenotype through the visualization of DNA profiles, and (3) acquire proficiency in determining DNA profiles and generating QR codes. Therefore, the novel training project greatly improved the teaching effectiveness of the hands-on experimental course and was more suitable for lesson teaching.

## Data Availability

The datasets generated and/or analysed during the current study are available in the Open Science Framework repository [https://osf.io/mywxa/]. Username is amoeba2000@126.com and the password is fjslsjs88216145.

## Abbreviations

STR: short tandem repeat
QR: quick response
TE: Tris-EDTA
MSDS: Material Safety Data Sheets
DNA: deoxyribonucleic acid
NIST: National Institute of Standards and Technology.
